# Scalable Synthesis of Aragonite Whiskers Under Higher Initial Ca^2+^ Concentrations

**DOI:** 10.3390/nano15241894

**Published:** 2025-12-17

**Authors:** Ruixue Wang, Zihao Xu, Baojun Yang, Bainian Wang

**Affiliations:** School of Chemistry and Chemical Engineering, Hefei University of Technology, Hefei 230009, China; 2023170876@mail.hfut.edu.cn (R.W.); 2024171027@mail.hfut.edu.cn (Z.X.)

**Keywords:** carbide slag, aragonite whiskers, scalable synthesis, resource recycling

## Abstract

Calcium carbonate (CaCO_3_) whiskers are promising materials for the high-value utilization of calcium-based resources. Here, aragonite whiskers were synthesized at a carbonation temperature of 90 °C using carbide slag ammonium leachate as the calcium source and CO_2_ as the precipitant. The effects of control agents, carbonation temperature, Ca^2+^ solution feeding rate, CO_2_ flow rate, and stirring speed on whisker morphology and aspect ratio were systematically investigated. Characterization via SEM and XRD revealed that the optimal conditions—carbonation temperature of 90 °C, Ca^2+^ feeding rate of 1.2 mL∙min^−1^, ethanol addition of 2 mL, CO_2_ flow rate of 150 mL∙min^−1^, and stirring speed of 300 rpm—yielded uniform CaCO_3_ whiskers with an average length of ~10 μm, an aspect ratio of ~24, and an aragonite purity of 99.42%. TEM confirmed that the whiskers are single crystals growing preferentially along the [001] direction. Hydroxyl groups were found to suppress lateral growth on the (200) facet, favoring elongation along the c-axis and enabling high-aspect-ratio whisker formation. These findings provide useful guidance for the scalable synthesis and industrial application of aragonite whiskers.

## 1. Introduction

Carbide slag, a solid waste byproduct of acetylene generation, contains approximately 90% Ca(OH)_2_ (dry basis) and represents a valuable calcium-rich resource [1]. Calcium carbonate (CaCO_3_), an important inorganic filler, is extensively used in the plastics, rubber, and papermaking industries [2]. CaCO_3_ exists in three polymorphic forms—calcite, aragonite, and vaterite—with relative stabilities in order of calcite > aragonite > vaterite [3]. Calcite generally exhibits a blocky or granular morphology, aragonite typically forms needle- or rod-like crystals, while vaterite tends to adopt spherical or disc-like structures [4]. By carefully modulating crystallization conditions, CaCO_3_ with tunable morphologies can be synthesized. The synthesis of aragonite whiskers is of great interest due to their superior performance as functional reinforcing fillers. Their needle-like monocrystalline structure and high aspect ratio enable them to effectively transfer stress within composite matrices, significantly enhancing mechanical properties such as tensile strength, modulus, and impact toughness in polymers (e.g., plastics, rubber) and cement [5]. Beyond these applications, they are also utilized in coatings, friction materials, and as asphalt modifiers to improve wear resistance and stability [6]. Consequently, developing routes to produce high-value-added aragonite whiskers from low-cost sources like carbide slag presents significant practical and environmental merits.

Several synthetic strategies have been established for aragonite whiskers, with hydrothermal synthesis [7], double decomposition [8], and carbonation [9] being the most widely adopted. These approaches generally rely on the interaction between control agents and crystal facets, which influence nucleation kinetics, guide oriented growth, and thus tailor whisker morphology [10]. For instance, Le et al. [11] employed calcium dodecyl benzenesulfonate as a control agent in a microreactor system, obtaining CaCO_3_ whiskers with an average length of ~27 μm, an aspect ratio of ~58, and an aragonite purity above 98%. Hua et al. [12] utilized Mg^2+^ as a control agent in a gas-liquid contact system, producing whiskers with lengths of ~20 μm, aspect ratios of 15–20, and a purity of 99.38%. Similarly, Meng et al. [13] synthesized CaCO_3_ whiskers via electrochemical cathodic reduction using calcium chloride, sodium bicarbonate, and magnesium chloride, yielding products with an average length of 18.78 μm, an aspect ratio of 36.82, and a purity of 94.59%. While traditional methods for synthesizing aragonite whiskers are well-established, they often rely on high-purity reagents and energy-intensive processes. A promising and innovative direction involves the use of secondary calcium sources, which not only reduces costs but also aligns with the principles of the circular economy. This approach treats calcium-rich industrial wastes as valuable feedstocks. For instance, yellow phosphorus slag [14] has been successfully converted into high-purity aragonite whiskers, and dolomite [12] has been utilized as a raw material for whisker production. Similarly, Hu et al. [15] demonstrated a novel reversible reaction pathway that synthesizes aragonite directly from ground calcium carbonate (GCC) in a MgCl_2_ solution, bypassing the high-energy calcination of limestone. These studies underscore that utilizing alternative calcium sources is a viable and advanced strategy for sustainable materials synthesis. In this context, our work utilizes carbide slag, another abundant calcium-rich waste, as a novel feedstock for the efficient production of aragonite whiskers, representing a significant contribution to this emerging field.

Because aragonite is a metastable phase, it readily transforms into the thermodynamically stable calcite during synthesis [16]. As a result, most reported methods employ relatively low initial Ca^2+^ concentration (e.g., 0.1 mol∙L^−1^) and rely on precise control of system supersaturation to stabilize aragonite and regulate whisker morphology [17,18,19]. However, these conditions typically result in low single-pass yields and high production costs, which hinder their industrial applicability.

To address these limitations, the present study investigates the carbonation synthesis of CaCO_3_ whiskers from carbide slag at a higher initial Ca^2+^ concentration (0.625 mol·L^−1^). By strategically selecting control agents and systematically optimizing process parameters, large-scale synthesis of well-defined aragonite whiskers was achieved under mild conditions. The resulting products exhibit uniform morphology, with an average length of ~10 μm, an aspect ratio of ~24, and an aragonite purity of 99.42%.

## 2. Materials and Methods

### 2.1. Materials

The carbide slag used in the experiment was provided by a certain enterprise. Additional characterization of the carbide slag, including XRD, SEM/EDS, and DTA/TG analyses, can be found in Figures S1 and S2. Its major components and chemical composition are shown in Table 1.

Ammonium chloride (NH_4_Cl, ≥99.5%), magnesium chloride (MgCl_2_, ≥98.0%), ammonium acetate (≥98.0%), aluminum chloride (AlCl_3_, ≥97.0%), anhydrous magnesium sulfate (MgSO_4_, ≥99.0%), zinc chloride (ZnCl_2_, ≥98.0%), sodium hexametaphosphate (≥95.0%), polyethylene glycol (PEG), tributyl phosphate (≥98.5%), pentaerythritol (≥98.0%), anhydrous ethanol (≥99.7%), triammonium phosphate (≥95.0%), sodium dodecyl sulfonate (SDS, ≥97.0%), glucose, triethanolamine, and ammonium bicarbonate (≥98.0%) were purchased from Sinopharm Chemical Reagent Co., Ltd., Shanghai, China. All reagents were not treated before use.

### 2.2. Preparation of CaCO_3_ Whiskers

**Figure 1:**
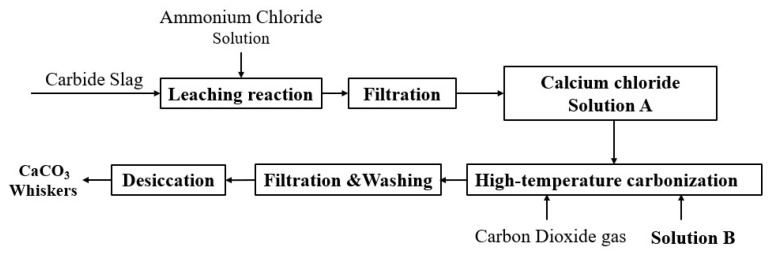
Schematic diagram of the preparation route of CaCO_3_ whiskers from carbide slag.

The experimental procedure followed the technical route outlined in Figure 1. Briefly, 10 g of carbide slag was added to 100 mL of 2.9 mol∙L^−1^ NH_4_Cl solution and leached at 40 °C for 1 h. After filtering to remove insoluble impurities, the resulting mother liquor was diluted with deionized water to obtain a Ca^2+^ solution (Solution A, 1.0 mol∙L^−1^). A predetermined amount of control agent was dissolved in 30 mL of deionized water to obtain Solution B. Under controlled temperature and stirring conditions (magnetic stirring), 50 mL of Solution A was pumped at a specified feeding rate into Solution B, which was placed in a specially designed reactor with good sealing, while CO_2_ gas was continuously introduced at a defined flow rate. Upon completion of adding Solution A, the CO_2_ flow was stopped. The resulting suspension was then filtered, washed, and dried to yield a white powder product for subsequent characterization.

### 2.3. Characterization

The as-prepared CaCO_3_ products were characterized via scanning electron microscopy (SEM, SU8020, Hitachi, Tokyo, Japan) operated at an accelerating voltage of less than or equal to 5 kV and transmission electron microscopy (TEM, JEM-2100F, JEOL, Tokyo, Japan) operated at an accelerating voltage of 60 kV. The X-ray diffraction (XRD, Rigaku D/MAX-2500VL/PC, Rigaku, Tokyo, Japan) patterns obtained on a DMAX-*γ* X-ray diffractometer using Cu Ka radiation were used to determine the identity of the crystalline phase. The functional groups of the samples were analyzed via Fourier transform infrared spectroscopy (FTIR, Nicolet 6700, Thermo Fisher, Waltham, MA, USA, infrared spectrometer, mid-infrared-ATR attenuated total reflection method, scanning wavelength range: 500–4000 cm^−1^).

## 3. Results and Discussion

**Figure 2:**
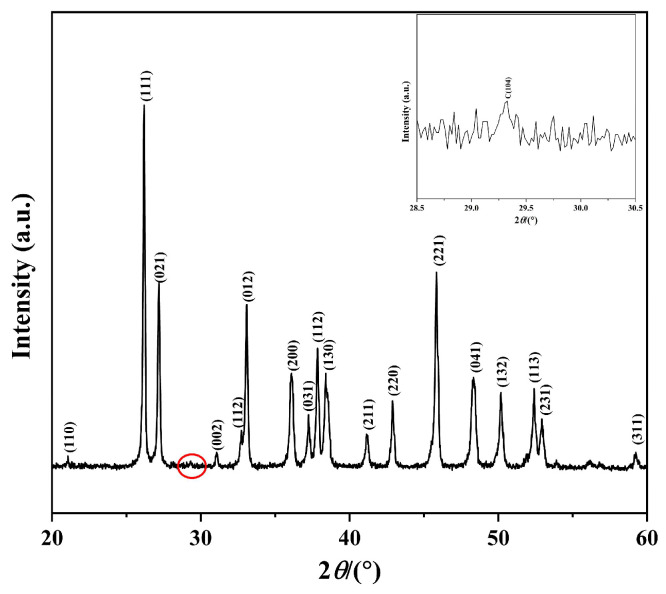
XRD pattern of the prepared CaCO_3_ sample. (The inset shows an enlarged view of the red circle region between 25° and 35°).

**Figure 3:**
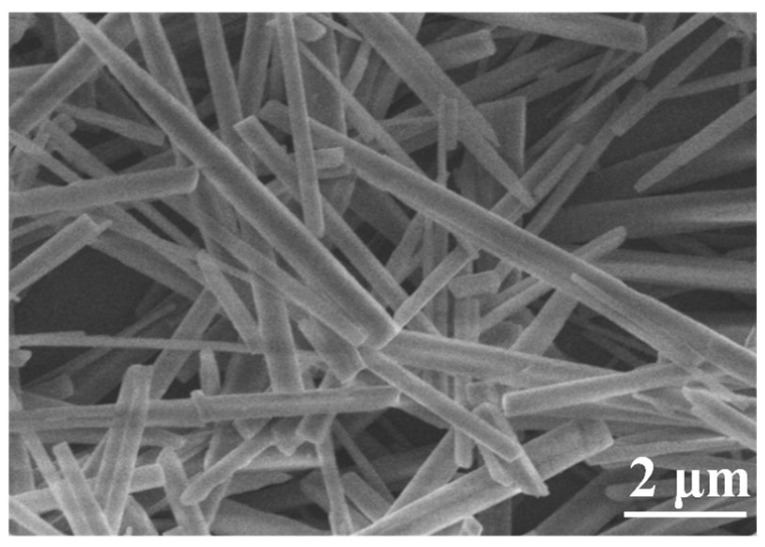
FE-SEM image of the prepared CaCO_3_ whiskers.

**Figure 4:**
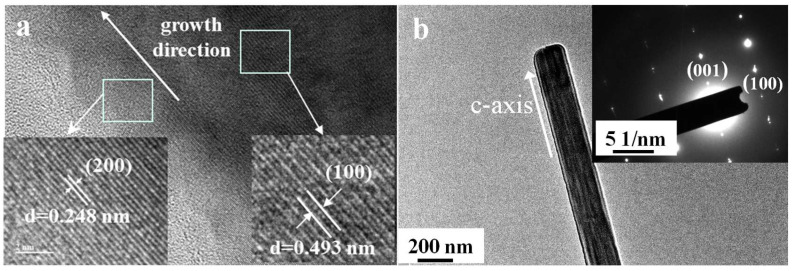
HR-TEM (**a**) and SAED (**b**) images of the prepared CaCO_3_ whiskers.

**Figure 5:**
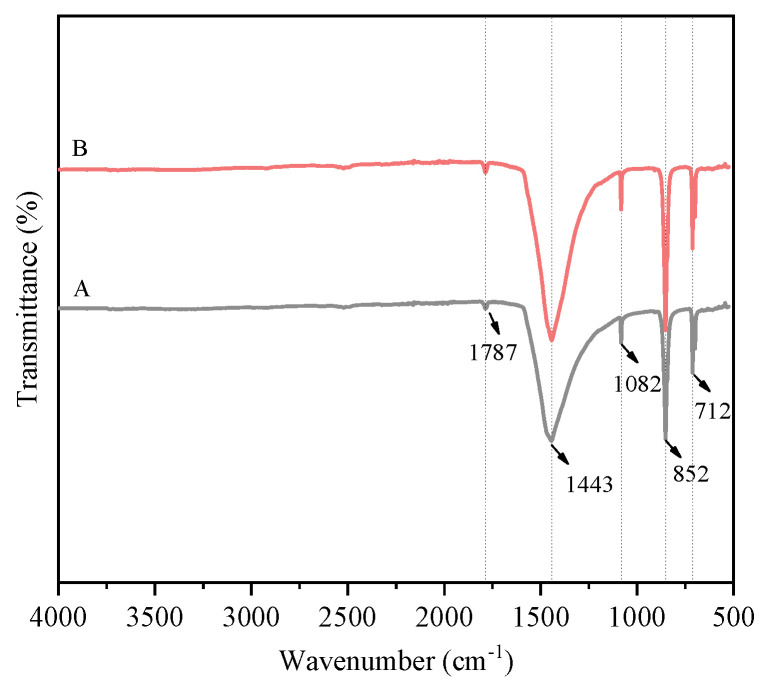
FT-IR spectrum of the prepared CaCO_3_ whisker sample. A—without the addition of ethanol; B—with the addition of 2 mL ethanol.

Figure 2, Figure 3, Figure 4 and Figure 5 show the XRD pattern, FE-SEM image, HR-TEM images, and FT-IR spectrum of CaCO_3_ samples prepared under the optimized synthesis conditions (carbonation temperature: 90 °C, Ca^2+^ solution feeding rate: 1.2 mL∙min^−1^, ethanol addition: 2 mL, CO_2_ flow rate: 150 mL∙min^−1^, stirring speed: 300 rpm).

As shown in Figure 2, the sharp diffraction peaks indicate the high crystallinity of the obtained CaCO_3_. All major diffraction peaks can be unambiguously indexed to orthorhombic aragonite CaCO_3_ (JCPDS 75-2230). Additionally, very weak reflections corresponding to the (104) facet of trigonal calcite CaCO_3_ (JCPDS 47-1743) are observed, as highlighted in the inset. Using Equation (1) [20,21], the aragonite content of the sample was calculated to be 99.42%.

(1) $$y=1-\frac{1}{1+\frac{3.9I_{A}}{I_{C}}}$$

where y (%) is the molar fraction of aragonite, *I_A_* is the integral intensity of the strongest (111) reflection of aragonite, and *I_C_* is the integral intensity of the strongest (104) reflection of calcite.

As observed in Figure 3, the CaCO_3_ whiskers exhibit smooth surfaces and well-defined morphologies, with an average diameter of ~200 nm and an aspect ratio of ~24.

The high-resolution TEM (HR-TEM) image (Figure 4a) reveals distinct lattice fringes with measured spacings of 0.248 nm and 0.493 nm (inset), corresponding to the (100) and (200) facets of aragonite, respectively (d_200_ = 0.24805 nm). This confirms that the lateral surfaces of the whiskers are dominated by the (200) facets. The selected area electron diffraction (SAED) pattern displays sharp and regularly arranged diffraction spots, indicating the single-crystal nature of the whiskers with an orthorhombic structure and a preferred growth orientation along the [001] c-axis.

In Figure 5, the absorption band observed at 1787 cm^−1^ is assigned to the C=O stretching vibration. Peaks at 1443 cm^−1^ and 1082 cm^−1^ are attributed to the asymmetric and symmetric C–O stretching vibrations, respectively. The band at 852 cm^−1^ is attributed to the out-of-plane deformation vibration of the ${\mathrm{CO}}_{3}^{2-}$ group, while the peak at 712 cm^−1^ arises from its in-plane deformation [22,23]. Comparison of spectra indicates no significant shifts or intensity changes in the absorption peaks with the addition of the control agent. Furthermore, no characteristic ethanol absorption bands were detected, likely due to its high solubility in water, low dosage, and effective removal during subsequent washing.

These results highlight that the appropriate selection of control agents, together with precise regulation of carbonation parameters, play a critical role in stabilizing aragonite formation and ensuring the morphological uniformity of the resulting whiskers.

### 3.1. Screening of Control Agents

**Figure 6:**
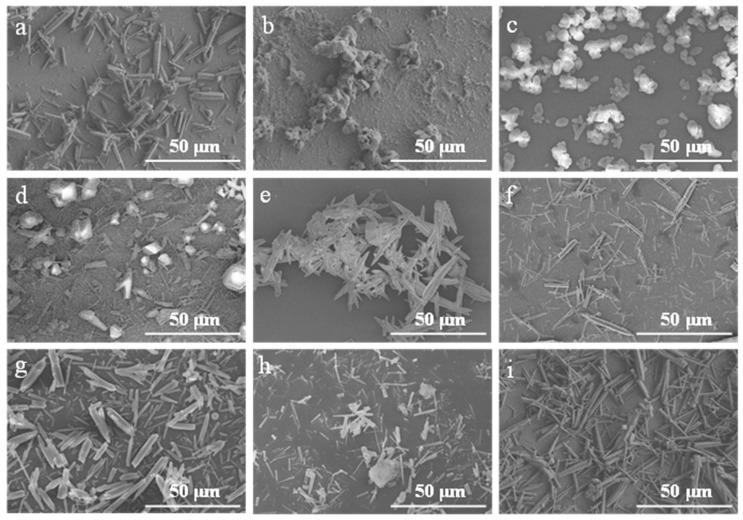
FE-SEM images of CaCO_3_ samples prepared with different control agents. (**a**) Blank; (**b**) Sodium hexametaphosphate; (**c**) Sodium dihydrogen phosphate; (**d**) PEG; (**e**) Tributyl phosphate; (**f**) Ethanol; (**g**) MgCl_2_; (**h**) Ammonium acetate; (**i**) Triethanolamine.

Under the experimental conditions of a Ca^2+^ solution feeding rate of 0.9 mL∙min^−1^, carbonation temperature of 90 °C, control agent addition 3% (solid, relative to the theoretical yield of CaCO_3_ by mass) or 1 mL (liquid), CO_2_ flow rate of 100 mL∙min^−1^, and stirring speed of 200 rpm, the morphology and aspect ratio of CaCO_3_ samples prepared with different control agents are summarized in Table 2. The control agents listed in Table 2 were selected based on their reported influence on crystallization processes, including inorganic ions (e.g., Mg^2+^, PO_4_^3−^), organic modifiers (e.g., glucose, triethanolamine), and polymers, to allow systematic investigation of their effects on CaCO_3_ morphology. Representative FE-SEM images of these samples are presented in Figure 6.

As summarized in Table 2 and illustrated in Figure 6, the CaCO_3_ samples synthesized without a control agent (Figure 6a) predominantly exhibited irregular, short rod-like morphologies. In contrast, the addition of various control agents led to notable changes in crystal morphology. Among them, the use of 1 mL ethanol (Figure 6f) yielded CaCO_3_ whiskers with elongated rod-like structures and improved dimensional uniformity. Quantitative analysis using Nano-Measure software (version 1.2) determined an average aspect ratio of ~13.5 for these whiskers. Based on these results, ethanol was identified as the most effective control agent under the present experimental conditions.

### 3.2. Effect of Carbonization Temperature and Ethanol Addition on the Crystal Phase of CaCO_3_

**Figure 7:**
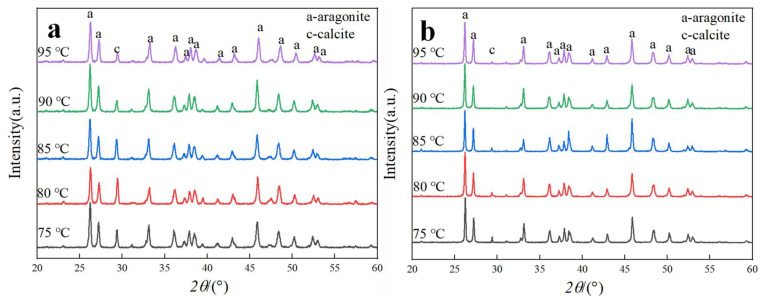
XRD patterns of CaCO_3_ samples prepared at different carbonation temperatures with and without ethanol. (**a**) Without ethanol. (**b**) With 1 mL ethanol.

**Figure 8:**
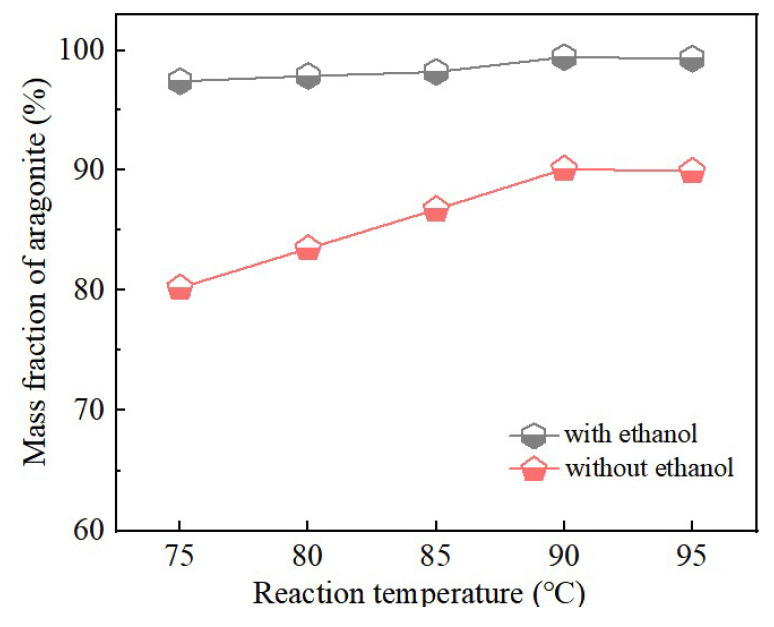
Aragonite CaCO_3_ samples were prepared at different carbonation temperatures with and without ethanol.

Carbonation temperature was a key parameter governing the crystalline phase of CaCO_3_ [24]. Even with constant synthesis conditions, variations in temperature can induce significant phase transformations. In this study, under fixed conditions (Ca^2+^ solution feeding rate: 0.9 mL∙min^−1^, CO_2_ flow rate: 100 mL∙min^−1^, stirring speed: 200 rpm), aragonite CaCO_3_ samples were synthesized at carbonation temperatures of 75, 80, 85, 90, and 95 °C, both without ethanol and with the addition of 1 mL ethanol. The results were summarized in Figure 7 and Figure 8.

Figure 7 and Figure 8 present the XRD patterns and aragonite content of CaCO_3_ samples synthesized at various carbonation temperatures, both in the absence and presence of ethanol. As shown in Figure 7, all samples within the investigated temperature range consisted of a mixture of aragonite and calcite, with aragonite constituting the predominant crystalline phase.

Figure 8 further demonstrated that the addition of ethanol markedly enhances aragonite formation compared with samples synthesized without ethanol. In the absence of ethanol, the aragonite content gradually increased with temperature up to 90 °C, where the maximum value was obtained. Further increases in carbonation temperature beyond 90 °C produced no substantial change in aragonite content. These results identify 90 °C as the optimal carbonation temperature under the present experimental conditions.

### 3.3. Effects of Operation Conditions on the Morphology of CaCO_3_

#### 3.3.1. Ca^2+^ Solution Feeding Rate

**Figure 9:**
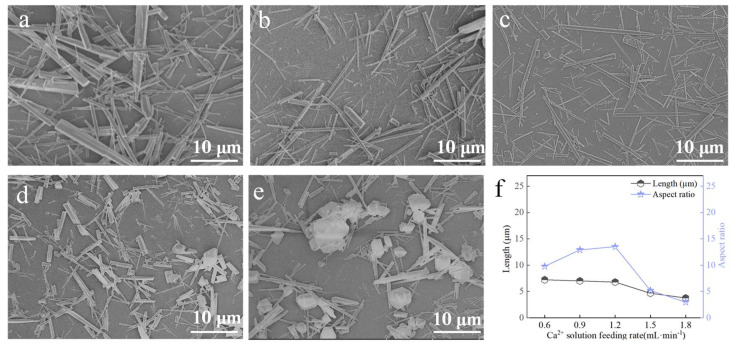
Effect of Ca^2+^ solution feeding rate on CaCO_3_ whiskers morphology ((**a**) 0.6 mL∙min^−1^; (**b**) 0.9 mL∙min^−1^; (**c**) 1.2 mL∙min^−1^; (**d**) 1.5 mL∙min^−1^; (**e**) 1.8 mL∙min^−1^) and the length/aspect ratio of the prepared whiskers (**f**).

At a carbonation temperature of 90 °C, with 1 mL ethanol, a CO_2_ flow rate of 100 mL∙min^−1^, and a stirring rate of 200 rpm, the influence of Ca^2+^ solution feeding rate on the morphology of CaCO_3_ whiskers was investigated (Figure 9). When the feeding rate was 0.6 mL∙min^−1^, the products were mainly thick rods with irregular morphologies. As the feeding rate increased, whisker diameters decreased and aspect ratios increased. At 1.2 mL∙min^−1^, the maximum aspect ratio was achieved, accompanied by improved morphological uniformity (Figure 9c,f). Beyond this point, higher feeding rates led to a sharp decrease in aspect ratio, and the products shifted toward irregular rods and block-like particles. Therefore, 1.2 mL∙min^−1^ was identified as the optimal Ca^2+^ solution feeding rate.

#### 3.3.2. Carbonation Temperature

**Figure 10:**
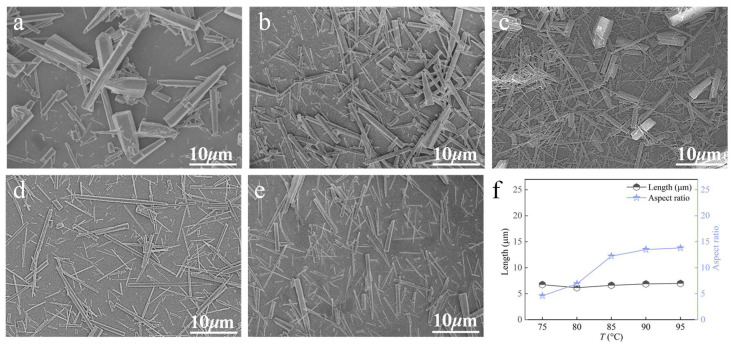
Effect of carbonation temperature on CaCO_3_ whiskers morphology ((**a**) 75 °C; (**b**) 80 °C; (**c**) 85 °C; (**d**) 90 °C; (**e**) 95 °C) and the length/aspect ratio of the prepared whiskers (**f**).

Carbonation temperature is the critical factor governing the crystal phase selection of the product, as it regulates the reaction kinetics and thermodynamics, the solution’s supersaturation, and even the crystal growth orientation [6,25]. At a Ca^2+^ solution feeding rate of 1.2 mL∙min^−1^, a CO_2_ flow rate of 100 mL∙min^−1^, and a stirring speed of 200 rpm, the influence of carbonation temperature was examined (Figure 10). When the temperature is below 90 °C, the products are mainly block-like and rod-like. As the temperature increases, whisker content in the product increases, their aspect ratio increases, and they become more uniform. When the temperature is further raised, no significant changes are observed in either the whisker content or the aspect ratio. Therefore, 90 °C is selected as the optimal temperature for the preparation of CaCO_3_ whiskers.

#### 3.3.3. Ethanol Addition

**Figure 11:**
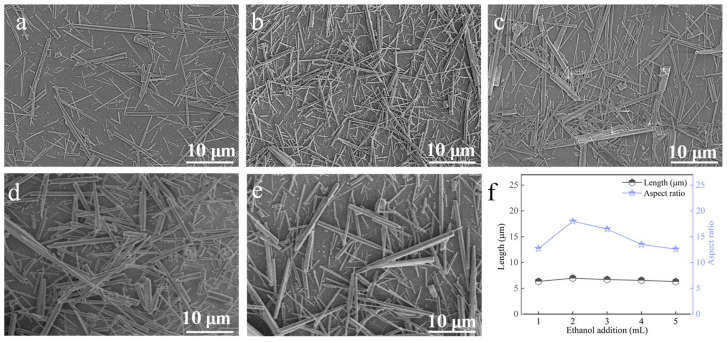
Effect of ethanol addition on CaCO_3_ whiskers morphology ((**a**) 1 mL; (**b**) 2 mL; (**c**) 3 mL; (**d**) 4 mL; (**e**) 5 mL) and the length/aspect ratio of the prepared whiskers (**f**).

At a carbonation temperature of 90 °C, with a Ca^2+^ solution feeding rate of 1.2 mL∙min^−1^, a CO_2_ flow rate of 100 mL∙min^−1^, and a stirring speed of 200 rpm, the influence of ethanol dosage was examined (Figure 11). With 1 mL of ethanol, the sample contained numerous dispersed fine whiskers. Increasing the ethanol amount to 2 mL yielded whiskers with the highest aspect ratio and improved dimensional uniformity (Figure 11b,f). Further increases in ethanol addition resulted in thicker whiskers and reduced aspect ratios. Hence, 2 mL of ethanol was determined as the optimal addition.

#### 3.3.4. CO_2_ Flow Rate

**Figure 12:**
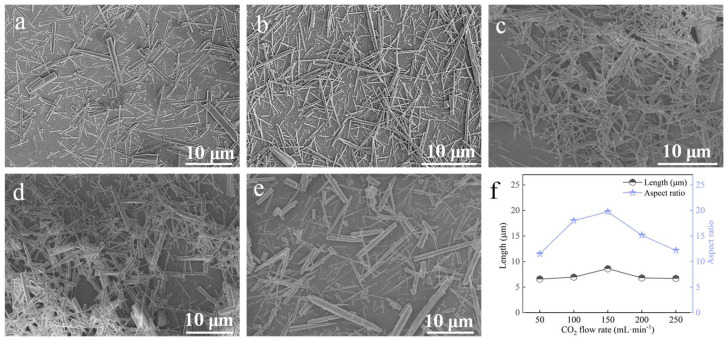
Effect of CO_2_ flow rate on CaCO_3_ whiskers morphology ((**a**) 50 mL∙min^−1^; (**b**) 100 mL∙min^−1^; (**c**) 150 mL∙min^−1^; (**d**) 200 mL∙min^−1^; (**e**) 250 mL∙min^−1^) and the length/aspect ratio of the prepared whiskers (**f**).

At a carbonation temperature of 90 °C, with a Ca^2+^ solution feeding rate of 1.2 mL∙min^−1^, ethanol addition of 2 mL, and a stirring speed of 200 rpm, the effect of CO_2_ flow rate was studied (Figure 12). At 50 and 100 mL∙min^−1^, the samples contained a high proportion of finer whiskers. Increasing the CO_2_ flow rate to 150 mL∙min^−1^ maximized the aspect ratio (Figure 12c,f). Further increases in flow rate caused whisker diameters to increase and aspect ratios to decrease. Therefore, 150 mL·min^−1^ was identified as the optimal CO_2_ flow rate.

#### 3.3.5. Stirring Speed (MAGNETIC Stirring)

**Figure 13:**
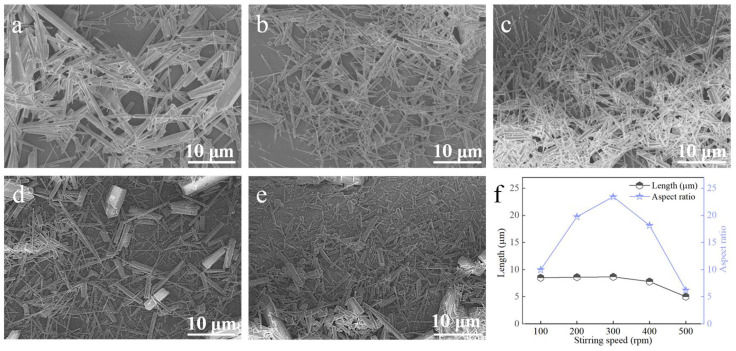
Effect of stirring speed on CaCO_3_ whiskers morphology ((**a**) 100 rpm; (**b**) 200 rpm; (**c**) 300 rpm; (**d**) 400 rpm; (**e**) 500 rpm) and the length/aspect ratio of the prepared whiskers (**f**).

At a carbonation temperature of 90 °C, with a Ca^2+^ solution feeding rate of 1.2 mL∙min^−1^, ethanol addition of 2 mL, and a CO_2_ flow rate of 150 mL∙min^−1^, the effect of stirring speed was investigated (Figure 13). When the stirring speed was below 300 rpm, the aspect ratio increased with increasing speed. At 300 rpm, the maximum aspect ratio was achieved (see Figure 13b,f). Further increases above 300 rpm reduced the aspect ratio. The observed trend can be explained by the interplay between mixing efficiency and shear stress. At stirring speeds below 300 rpm, the mass transfer rate is insufficient, leading to non-uniform mixing of Ca^2+^, CO_2_, and ethanol. This creates local concentration gradients that hinder the regular, oriented growth necessary for achieving a high aspect ratio. Consequently, the aspect ratio increases with improved mixing as the stirring speed rises to 300 rpm. Beyond this optimal point, however, excessive shear forces become dominant. These forces can fracture the elongated crystals or disrupt their directional alignment along the preferred crystallographic axis, thereby reducing the overall aspect ratio [26,27]. Therefore, 300 rpm was determined as the optimal stirring speed.

## 4. Formation Mechanism of Aragonite Whiskers

According to Ostwald’s rule of stages, crystallization occurs via two successive steps: nucleation and crystal growth. Nuclei first form in a supersaturated solution, after which ions continuously deposit onto these nuclei in accordance with crystallographic symmetry, replicating the unit cell structure and facilitating crystal growth [28,29]. Based on present experimental results, the formation mechanism of aragonite whiskers can be understood from two aspects.

### 4.1. Formation of Aragonite

The crystallization of aragonite is governed by the interplay of thermodynamic and kinetic factors. As a metastable phase, aragonite typically requires external intervention to compete against the thermodynamically favored calcite [30]. In this context, both carbonation temperature and ethanol addition play pivotal roles. As illustrated in Figure 7 and Figure 8, elevating carbonation temperature facilitates the transition from calcite to aragonite, indicating that temperature modulates the kinetics and supersaturation of CaCO_3_ polymorphs, thereby influencing phase selection. Concurrently, ethanol molecules act as an effective control agent by selectively adsorbing on specific crystallographic planes. This adsorption is mediated by the alcoholic hydroxyl (–OH) functional group, which binds to the calcite (104) facet through a dual mechanism: hydrogen bonding between the –OH group and oxygen atoms of surface ${\mathrm{CO}}_{3}^{2-}$, and coordination of the ethanol oxygen lone pairs with exposed Ca^2+^ ions [31,32,33]. Together, these interactions form a stable and ordered adsorption layer that inhibits the nucleation and growth of calcite, thereby favoring the alternative formation of aragonite.

The critical role of the hydroxyl group and the influence of molecular structure are further supported by control experiments with alternative additives. Molecules bearing multiple, accessible hydroxyl groups within a compact and rigid structure—such as triethanolamine and pentaerythritol—exhibited efficacy comparable to ethanol in promoting aragonite whiskers. In contrast, the long-chain and flexible polyethylene glycol (PEG) proved ineffective, yielding mostly aggregated particles (as shown in Figure S3). This contrast highlights that effective polymorph control requires not only the presence of hydroxyl groups but also a molecular geometry conducive to forming a dense, well-ordered inhibitory layer on the specific calcite surface.

**Figure 14:**
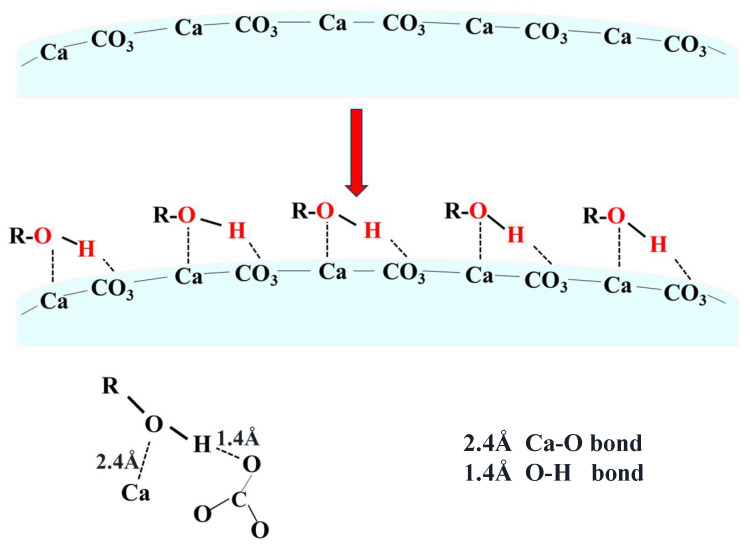
Schematic diagram of ethanol interaction with the calcite (104) facet (Ca-O and O-H bonds).

A schematic representation of this hydroxyl-mediated interaction is illustrated in Figure 14. This mechanism is consistent with the quantitative results in Figure 8, where the aragonite content in ethanol-assisted samples is significantly higher than in samples synthesized without ethanol, confirming the selective inhibition of calcite by hydroxyl-functionalized molecules.

### 4.2. Formation of CaCO_3_ Whiskers

Crystal growth is inherently anisotropic, with preferential extension along specific crystallographic orientations, giving rise to distinct morphologies [34]. In this study, ethanol plays a decisive role in directing the anisotropic growth of aragonite whiskers.

**Figure 15:**
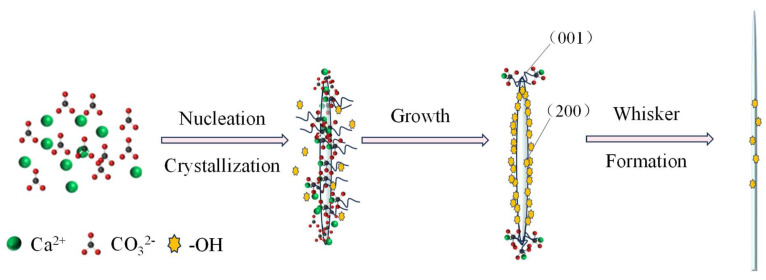
Schematic diagram of the ethanol-mediated growth mechanism of aragonite whiskers.

As illustrated in Figure 15, hydroxyl groups from ethanol are dynamically adsorbed onto the low-energy (200) facets, which correspond to the lateral surfaces of the whiskers (Figure 4). This adsorption inhibits lateral growth, while Ca^2+^ and ${\mathrm{CO}}_{3}^{2-}$ ions preferentially deposit on the higher-energy (001) facet, promoting elongation along the c-axis. This anisotropic deposition drives preferential extension along [001], resulting in whiskers with high aspect ratios and uniform morphologies.

## 5. Conclusions

Aragonite whiskers were successfully synthesized via a carbonation route at 90 °C using carbide slag as the calcium source, ammonium chloride as the leaching agent, and CO_2_ as the precipitant. The effects of carbonation temperature, ethanol addition, Ca^2+^ solution feeding rate, CO_2_ flow rate, and stirring speed on whisker morphology and aspect ratio were systematically evaluated. The optimal conditions were determined as 90 °C, 2 mL ethanol, a Ca^2+^ solution feeding rate of 1.2 mL∙min^−1^, a CO_2_ flow rate of 150 mL∙min^−1^, and a stirring speed of 300 rpm.

The results indicate that these parameters strongly influence whisker morphology and dimensional uniformity. Mechanistic analysis indicates that ethanol plays a decisive role in stabilizing aragonite and directing anisotropic growth. Ethanol molecules selectively adsorb onto the (104) facet of calcite, suppressing its growth, while hydroxyl groups adsorb on the (200) facet of aragonite whiskers, inhibiting lateral growth. This facet-selective adsorption promotes elongation along the c-axis, enabling the formation of aragonite whiskers with a high aspect ratio.

## Figures and Tables

**Table 1:** Major components and chemical composition of carbide slag.

Composition	CaO	SiO_2_	Al_2_O_3_	Na_2_O	MgO	Fe_2_O_3_	Others
Content (wt.%)	70.05	3.85	3.64	2.73	1.84	0.96	16.93

**Table 2:** Morphology and aspect ratio of CaCO_3_ samples prepared with different control agents.

No.	Control Agents	Morphology of CaCO_3_	Aspect Ratio
1	Blank	Irregular short rod-like	8.4
2	Ammonium hydrogen carbonate	Stacked flakes	—
3	Sodium hexametaphosphate	Blocky and small granules	—
4	Sodium dihydrogen phosphate	Elliptical	—
5	PEG	Irregular blocky, rod-like, and a Small amount of needle-like	—
6	D-(+)-Glucose	Block-shaped and spherical	—
7	Tributyl phosphate	Aggregated rods	6.4
8	Ethanol	Rod and whisker	13.5
9	SDS	Irregularly shaped sphere	—
10	AlCl_3_	Rod-like and needle-like	8.5
11	MgCl_2_	Rod-like and needle-like	8.6
12	MgSO_4_	Flaky and granular	—
13	Ammonium acetate	Rod-like and small amounts of whiskers	8.7
14	Triammonium phosphate trihydrate	Irregular block-like	—
15	Triethanolamine	Rod and whisker	10.2
16	ZnCl_2_	Particles and short rods	4.0
17	Pentaerythritol	Short rod-like	7.7

## Data Availability

All experimental datasets supporting the findings of this study are fully available within the manuscript.

## Supplementary Materials

The following supporting information can be downloaded at https://www.mdpi.com/article/10.3390/nano15241894/s1, Figure S1. SEM/EDS image of carbide slag. Figure S2. XRD pattern and DTA/TG image of carbide slag. Figure S3. FE-SEM images of CaCO_3_ samples prepared with (a) Triethanolamine; (b) pentaerythritol; (c) PEG.
