# Supplementary material for: Scalable Synthesis of Aragonite Whiskers Under Higher Initial Ca2+ Concentrations
Source: Nanomaterials (Basel). 2025 Dec 17;15(24):1894. doi: 10.3390/nano15241894 (PMC12736146; doi:10.3390/nano15241894)
Supplement: Supplementary file 1 [file nanomaterials-15-01894-s001.zip › nanomaterials-4004565-supplementary.pdf]

Supporting Information

Scalable synthesis of aragonite $CaCO_3$ whiskers under higher initial Ca^{2+} concentrations

Ruixue Wang[1], Zihao Xu[1], Baojun Yang[1]*, Bainian Wang[1]*

1 School of Chemistry and Chemical Engineering, Hefei University of Technology, Hefei 230009, China; 2023170876@mail.hfut.edu.cn; 2024171027@mail.hfut.edu.cn

* Correspondence: bj_yang@hfut.edu.cn; 200280068@hfut.edu.cn

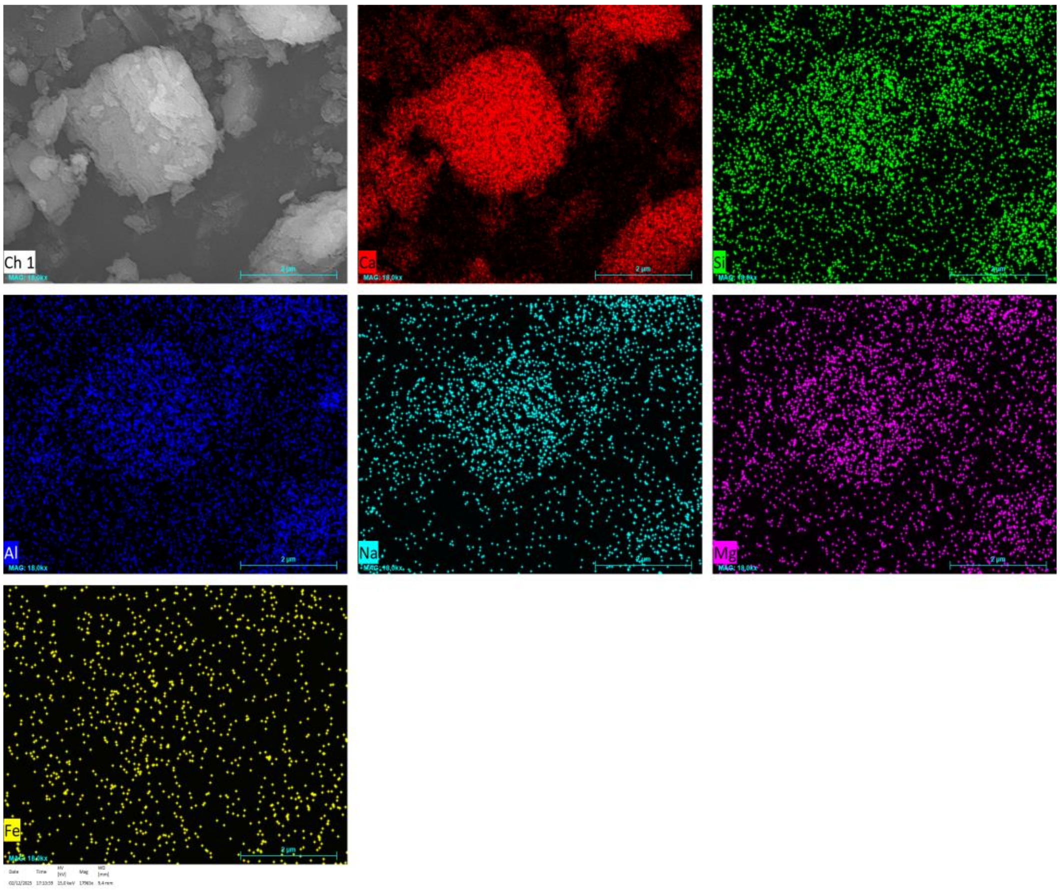

Figure S1. SEM/EDS image of carbide slag.

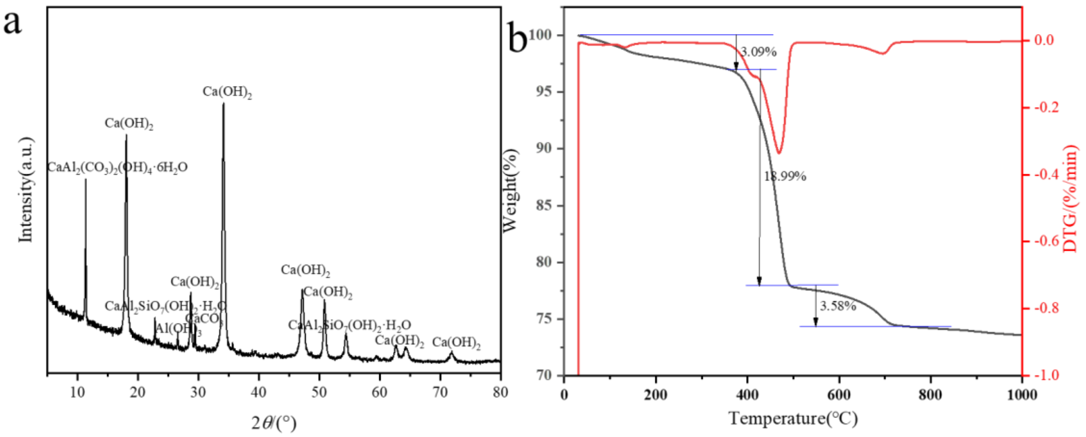

Figure S2. XRD pattern and DTA/TG image of carbide slag.

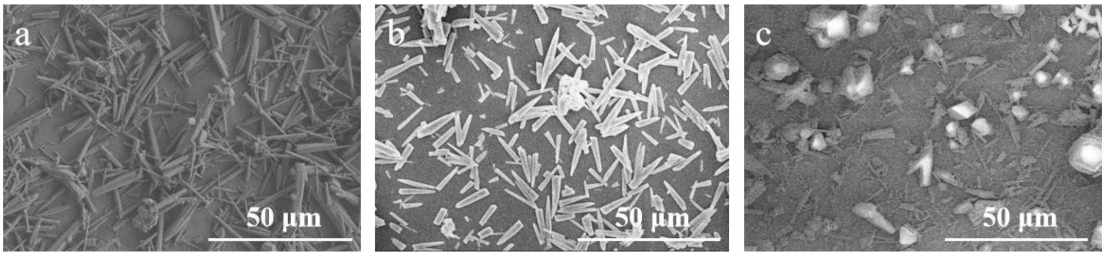

Figure S3. FE-SEM images of $CaCO_3$ samples prepared with a-Triethanolamine; b-pentaerythritol; c- PEG.
